# D-Mannose Inhibits Adipogenic Differentiation of Adipose Tissue-Derived Stem Cells *via* the miR669b/MAPK Pathway

**DOI:** 10.1155/2020/8866048

**Published:** 2020-12-10

**Authors:** Yitong Liu, Lijia Guo, Lei Hu, Chen Xie, Jingfei Fu, Yiyang Jiang, Nannan Han, Lu Jia, Yi Liu

**Affiliations:** ^1^Laboratory of Tissue Regeneration and Immunology and Department of Periodontics, Beijing Key Laboratory of Tooth Regeneration and Function Reconstruction, School of Stomatology, Capital Medical University, China; ^2^Department of Orthodontics School of Stomatology, Capital Medical University, China

## Abstract

The adipogenic differentiation of adipose tissue-derived stem cells (ADSCs) plays an important role in the process of obesity and host metabolism. D-Mannose shows a potential regulating function for fat tissue expansion and glucose metabolism. To explore the mechanisms through which D-mannose affects the adipogenic differentiation of adipose-derived stem cells *in vitro*, we cultured the ADSCs with adipogenic medium inducement containing D-mannose or glucose as the control. The adipogenic differentiation specific markers *Pparg* and *Fabp4* were determined by real-time PCR. The Oil Red O staining was applied to measure the lipid accumulation. To further explore the mechanisms, microarray analysis was performed to detect the differences between glucose-treated ADSCs (G-ADSCs) and D-mannose-treated ADSCs (M-ADSCs) in the gene expression level. The microarray data were further analyzed by a Venn diagram and Gene Set Enrichment Analysis (GSEA). MicroRNA inhibitor transfection was used to confirm the role of key microRNA. *Results*. D-Mannose intervention significantly inhibited the adipogenic differentiation of ADSCs, compared with the glucose intervention. Microarray showed that D-mannose increased the expression of miR669b, which was an inhibitor of adipogenesis. In addition, GSEA and western blot suggested that D-mannose suppressed the adipogenic differentiation *via* inhibiting the MAPK pathway and further inhibited the expression of proteins related to glucose metabolism and tumorigenesis. *Conclusion*. D-Mannose inhibits adipogenic differentiation of ADSCs *via* the miR669b/MAPK signaling pathway and may be further involved in the regulation of glucose metabolism and the inhibition of tumorigenesis.

## 1. Introduction

The prevalence of obesity is constantly increasing and poses a global challenge [1], which is associated with many diseases [2–4]. Central deposition of adipose tissue in individuals increases the risk of cardiovascular disease and mortality [5, 6] and the risk of developing type 2 diabetes mellitus (T2DM) ascribed to insulin resistance and dysfunction of pancreatic islet *β*-cells [7]. Obesity arises from adipocyte hypertrophy and hyperplasia due to impaired regulation of adipogenesis and an imbalance between lipolysis and lipogenesis, ultimately resulting in the expansion of fat mass [8]. It is accompanied by adipocyte turnover, which involves the expansion and differentiation of adipose tissue-derived stem cells (ADSCs) [9]. During this process, peroxidase proliferator-activated receptor-g (*Pparg*) acts in concert to regulate the adipocyte differentiation program, coordinately driving the expression of adipocyte-specific proteins at the final stages of differentiation, such as adipocyte fatty acid-binding protein 4 (*Fabp4*) [8]. Therefore, *Pparg* and *Fabp4* can be used as important markers for assessing the adipogenic differentiation effect on ADSCs.

D-Mannose is an epimer of D-glucose, which exists naturally in many kinds of plants and fruits, and is a popular nutritional and health-beneficial food supplement worldwide [10]. Previous studies have demonstrated that D-mannose showed a positive impact on maintaining good health. For instance, D-mannose exhibits strong anti-inflammatory properties and prevents recurrent urinary tract infection in humans, with fewer side effects [11]. Also, other studies confirmed that D-mannose showed the function of immune regulation. A study by Zhang et al. showed that D-mannose increased the percentage of regulatory T cells and thus suppressed immunopathology in mouse models of airway inflammation and autoimmune diabetes [12], and our previous study showed that D-mannose enhanced the immunomodulation function of periodontal ligament stem cells on T cells *via* inhibiting interleukin- (IL-) 6 secretion [13]. Besides, D-mannose may be involved in the regulation process of glucose metabolism and fat tissue expansion. Sharma revealed that D-mannose could prevent diet-induced obesity and improve host metabolism [14]. Moreover, Zhang et al. reported that supplementation of 25 mM D-mannose *in vitro* or 1.1 M D-mannose in drinking water *in vivo* could suppress type 1 diabetes in mice [12]. These results suggest that D-mannose appears to regulate adipogenic differentiation, but this conclusion has not been verified, and the related molecular mechanism remains unclear.

This study was aimed at evaluating the effect of D-mannose supplementation on the adipogenic differentiation function of mouse adipose-derived stem cells (ADSCs) and at exploring the underlying mechanism *in vitro.*

## 2. Materials and Methods

### 2.1. Cell Culture

Mouse adipose-derived stem cells (ADSCs) were isolated from visceral fat tissues near the epidermis. Briefly, we obtained the abdominal adipose tissue from 8-week-old C57BL/6 mice and placed it in sterile Petri dishes with phosphate-buffered saline (PBS, Sigma-Aldrich, St. Louis, MO, USA) inside. After adequate mincing and washing in Hank's solution containing collagenase type II (Sigma-Aldrich, St. Louis, MO, USA), the tissues were digested at 37°C for 30-60 min until a smooth and even consistency resulted. Then, cells were isolated *via* centrifugation and filtered using a 70 *μ*m nylon mesh, incubated with erythrocyte lysis buffer (150 mM NH_4_Cl, 10 mM KHCO_3_, and 0.1 mM EDTA), refiltered using a 40 *μ*m cell strainer, and eventually resuspended in complete medium composed of DMEM supplemented with 10% fetal bovine serum (FBS, Equitech-Bio, Kerrville, TX), 5% Pen/Strep (Biofluids, Inc.), and 5% glutamine (Invitrogen). Cultures were incubated at 37°C, with 5% CO_2_, in a humidified atmosphere. The cell medium was changed every 2-3 days. Cells were passaged when they became 70% to 80% confluent. All cells used in this study were of passage 2.

### 2.2. Osteogenic Differentiation Assay under Glucose or D-Mannose Treatment

ADSCs were seeded into 6-well plates at a density of 4 × 10^5^ cells/cm^2^ and incubated overnight in culture medium at 37°C and 5% CO_2_. After that, cells were divided into two groups. Cells in the glucose group (G-ADSCs) were cultured in osteogenic medium with 25 mM glucose treatment which referred to the glucose contained in the DMEM medium, and cells in the D-mannose group (M-ADSCs) were cultured in glucose-free osteogenic medium with 25 mM D-mannose (Sigma-Aldrich, St. Louis, MO, USA) treatment. Besides that, the osteogenic medium contained 2 mM *β*-glycerophosphate (Sigma-Aldrich, St. Louis, MO), 10 nM dexamethasone (Sigma-Aldrich, St. Louis, MO), and 100 *μ*M L-ascorbic acid 2-phosphate (Wako Chemicals USA, Richmond, VA). All the medium preparation methods are based on the methods in [12, 15] and our previous studies [13]. Total RNA was extracted from each group with the TRIzol reagent (Invitrogen, Carlsbad, CA, USA) after ten days of inducement, and the gene expression levels of *Bglap* and *Alpl* were assayed by real-time PCR analysis.

### 2.3. Adipogenic Differentiation Assay under Glucose or D-Mannose Treatment

ADSCs were seeded into 6-well plates at a density of 4 × 10^5^ cells/cm^2^ and incubated overnight in culture medium at 37°C and 5% CO_2_. After that, cells were divided into two groups. As mentioned above, cells in the glucose group (G-ADSCs) were cultured in adipogenic medium with 25 mM glucose treatment and cells in the D-mannose group (M-ADSCs) were cultured in adipogenic medium with 25 mM D-mannose treatment. The adipogenic medium contained 500 *μ*M isobutylmethylxanthine (Sigma-Aldrich, St. Louis, MO), 500 nM hydrocortisone (Sigma-Aldrich, St. Louis, MO), 60 *μ*M indomethacin (Sigma-Aldrich, St. Louis, MO), 100 *μ*M L-ascorbic acid 2-phosphate, and 10 *μ*g/ml insulin (Sigma-Aldrich, St. Louis, MO). The gene expressions of *Pparg* and *Fabp4* were analyzed *via* real-time PCR analysis after adipogenic induction.

### 2.4. Quantitative Real-Time PCR Analysis

After the osteogenic inducement, total RNA was extracted from each group. We synthesized cDNA from 2 *μ*g aliquots of total RNA, oligo(dT) (Invitrogen, Carlsbad, CA, USA), and RNaseOUT™ Recombinant Ribonuclease Inhibitor (Invitrogen, Carlsbad, CA, USA), according to the manufacturer's protocol. Real-time PCR reactions were performed with the Power SYBR® Green PCR Master Mix (Life Technologies, Warrington, UK) and primers that targeted *Bglap*, *Alpl*, *Pparg*, and *Fabp4* (Table 1).

### 2.5. Oil Red O Staining

After 14 days of adipogenic induction, the cells were fixed with 4% paraformaldehyde and with Oil Red O (Sigma-Aldrich, St. Louis, MO) according to the manufacturer's instructions. The lipid droplets were observed by microscopy in at least five random fields.

### 2.6. Microarray Analysis

MicroRNA expression profiles in G-ADSCs and M-ADSCs were checked using microarray analysis. ADSCs were cultured in glucose-free DMEM culture medium (10% FBS, 5% Pen/Strep, and 5% glutamine) supplemented with 25 mM D-mannose (M-ADSCs) or in the high-glucose (25 mM) DMEM culture medium with 10% FBS, 5% Pen/Strep, and 5% glutamine (G-ADSCs). Cells were harvested three days later and stored in RNAlater (Ambion) and shipped for microarray analysis. RNA was isolated using the RNeasy Mini kit (Qiagen) and quantified in a NanoDrop spectrophotometer. RNA sample purity ratios were more than 1.8 for ratios of 260/280 nm and 260/230 nm and RIN (RNA Integrity Number) values were greater than 7.8 as evaluated on Bioanalyzer 2100 (Agilent Technologies). For the microarray assay, samples were loaded in Affymetrix GeneChip® Microarrays and proceeded in GeneChip platform stations, and data were analyzed by using Affymetrix GeneChip Operating Software and RStudio.

### 2.7. Transfection of the miR669b-5p Inhibitor

The differential expression profiles of microRNA between G-ADSCs and M-ADSCs were detected by microRNA microarray, and miR669p was extremely significantly increased after D-mannose treatment. For further exploration, ADSCs were transfected with the miR669b-5p inhibitor. Briefly, ADSCs were seeded at a density of 2 × 10^5^ cells per well in 6-well plates in culture medium without antibiotics. Cells were transfected with a mission synthetic microRNA inhibitor, mmu-miR-669b-5p, and negative control (Sigma, USA) using the 4D-Nucleofector™ X Kit (Lonza) at 70% confluence following the manufacturer's instructions. Transfection efficiency was determined by quantitative real-time PCR at day 1 and day 2 after transfection.

The transfected ADSCs were induced in adipogenic medium. The gene expressions of *Pparg* and *Fabp4* were analyzed *via* real-time PCR analysis after adipogenic induction, and Oil Red O staining was applied after 14 days of adipogenic induction.

### 2.8. Detection of miR669b-5p Expression

Total RNA was extracted using the mirVana miRNA isolation kit (Applied Biosystems/Ambion, Austin, TX) according to the manufacturer's instructions. We converted total RNA to cDNA using a TaqMan microRNA Reverse Transcription kit (Applied Biosystems). Real-time PCR was carried out with the Power SYBR® Green PCR Master Mix (Life Technologies, Warrington, UK) as previously described, and RNU48 was used as an endogenous control.

### 2.9. Gene Set Enrichment Pathway Analysis (GSEA)

GSEA primarily analyzes microarray data, using genomic and genetic sequencing to detect significant biological differences in microarray datasets. In this study, differentially expressed genes and common crucial pathways between G-ADSCs and M-ADSCs from microarray data were identified by GSEA as previously described [16]. Briefly, computing and general statistical analysis were processed in the R computing language (http://www.R-project.org/) with the package GSEABase. The datasets were normalized, and the intensity of the log10 probe set was calculated using the robust multichip averaging algorithm with bioconductors. The selected differentially expressed genes were required to be mapped to an explicit Kyoto Encyclopedia of Genes and Genomes (KEGG; http://www.genome.jp/kegg/) pathway of the Database for Annotation, Visualization and Integrated Discovery (DAVID; http://david.abcc.ncifcrf.gov/) for further analysis using the Venn and meta-analysis methods. Pathway analysis of each dataset was performed independently. The variability was measured in the interquartile range (IQR), and a cut-off was set to foreclose IQR values < 0.5 for all the remaining genes. If one gene was targeted in multiple probe sets, the probe set with the greatest variability was retained. In addition, genes in each pathway were subjected to a statistical analysis system (SAS), and each pathway's *P* value was obtained in the permutation test with 1000x. *P* < 0.05 was considered to indicate a statistically significant difference.

### 2.10. Western Blot

Total protein was extracted with NE-PER nuclear and cytoplasmic extraction reagents (Thermo). Next, 50-100 *μ*g aliquots of protein was separated on 10% polyacrylamide-SDS gels (Pplygen) and transferred to Immobilon™-P membranes (Millipore). After blocking with TBS/5% nonfat dry milk (Pplygen) for 1 h, the membrane was incubated with antibodies against mouse JNK (1 : 1000, #9252, Cell Signaling Technology), phospho-JNK (1 : 1000, #9255, Cell Signaling Technology), ERK (1 : 1000, #4695, Cell Signaling Technology), phospho-ERK (1 : 1000, #4370, Cell Signaling Technology), p38 (1 : 1000, #8690, Cell Signaling Technology), phospho-p38 (1 : 1000, #9215, Cell Signaling Technology), insulin receptor alpha (1 : 1000, ab203037, Abcam), insulin receptor beta (1 : 1000, ab69508, Abcam), glucose transporter GLUT4 (1 : 1000, ab654, Abcam), c-Myc (1 : 1000, ab32072, Abcam), and Hsp 90 antibody (1 : 1000, #4875, Cell Signaling Technology) overnight at 4°C. Next, membranes were incubated with horseradish peroxidase-conjugated secondary antibodies (Pierce, Malibu, CA, USA) for 1 h at room temperature. Antibody binding was visualized with an enhanced chemiluminescence kit, according to the manufacturer's protocols (Pierce).

### 2.11. Statistical Analysis

All data were repeated in three to five independent experiments. Statistical analysis was performed using SPSS 16.0 software. Unless otherwise noted, statistical significance comparison was analyzed by two-tailed Student's *t*-test between two groups and by one-way ANOVA between more than two groups. *P* values less than 0.05 (*P* < 0.05) were determined to be statistically significant.

## 3. Results

### 3.1. D-Mannose Showed No Effect on Osteogenesis but Inhibited Adipogenic Differentiation of ADSCs

**Figure 1:**
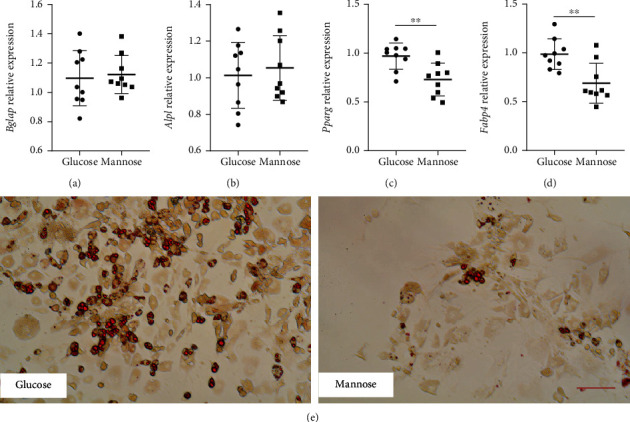


To examine the effects of D-mannose on ADSC differentiation, we induced ADSCs with osteogenic medium containing glucose or D-mannose for 7 days; then, the expression levels of the early osteogenic differentiation-specific markers *Bglap* and *Alpl* were identified by real-time PCR. The results showed that D-mannose-treated ADSCs had no difference compared with the glucose-treated group on osteogenic differentiation (Figures 1(a) and 1(b)). Meanwhile, we induced ADSCs with adipogenic medium containing glucose or D-mannose for 7 days, and the results of PCR showed that D-mannose treatment significantly decreased the expression of *Pparg* and *Fabp4* during adipogenic induction compared with the glucose-treated group (Figures 1(c) and 1(d)). Moreover, the Oil Red O staining confirmed that D-mannose decreased the lipid accumulation during the adipogenic induction in ADSCs (Figure 1(e)). These data indicated that D-mannose inhibited adipogenic differentiation function of ADSCs.

### 3.2. MicroRNA669b Was Significantly Upregulated by D-Mannose Intervention

**Figure 2:**
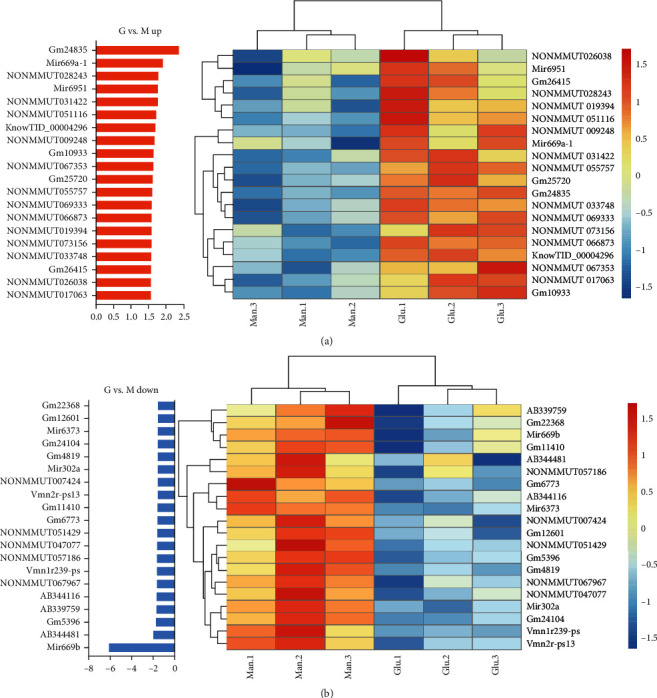


We then explored the mechanisms underlying D-mannose inhibition of ADSC adipogenic differentiation. Microarray analysis was performed to detect differences in gene expression levels between glucose-treated ADSCs (G-ADSCs) and D-mannose-treated ADSCs (M-ADSCs). Among the samples, miR669b was significantly upregulated by D-mannose compared to the other miRNAs (Figure 2(b)). Therefore, we hypothesized that miR669b may play a key role during the inhibition of adipogenic differentiation by D-mannose.

### 3.3. Anti-miR669b-5p Improved the Adipogenic Differentiation in ADSCs

**Figure 3:**
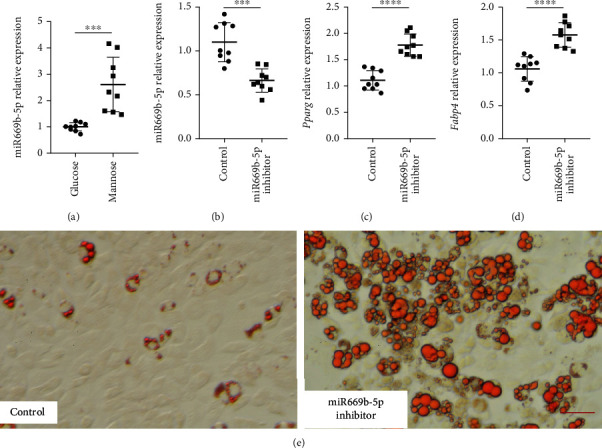


To verify the hypothesis, we need to clarify the role of miR669b in the adipogenic differentiation process. Real-time PCR was applied to investigate the expression of miR669b-5p under D-mannose intervention, and indeed, the result confirmed that the miR669b-5p expression level was significantly upregulated in the D-mannose group compared with the glucose group, indicating that D-mannose intervention activated miR669b expression in ADSCs (Figure 3(a)). To further explore the key role of miR669b-5p during the adipogenic differentiation in ADSCs, we transfected the cells with a microRNA inhibitor, mmu-miR-669b-5p, and negative control. 24 h after the transfection, the miR669b-5p level was significantly downregulated about 40% (Figure 3(b)). This result indicated that the level of miR669b-5p in ADSCs was manipulated using the miR669b-5p inhibitor.

We next performed the adipogenic induction using the transfected cells to determine if the adipogenic differentiation ability was mediated by miR669b-5p. After 7 days of induction, the gene expressions of *Pparg* and *Fabp4* were analyzed *via* real-time PCR analysis. The results showed that the anti-miR669b-5p inhibitor increased the expression levels of *Pparg* and *Fabp4* (Figures 3(c) and 3(d)). Oil Red O staining confirmed that anti-miR669b-5p increased the lipid accumulation during the adipogenic induction in ADSCs (Figure 3(e)). These data indicated that miR669b-5p could be an inhibitor of the adipogenic differentiation function of ADSCs, and D-mannose may inhibit adipogenic differentiation by upregulating the miR669b-5p expression.

### 3.4. MAPK Signaling Pathway Plays a Key Role during D-Mannose Regulation

**Figure 4:**
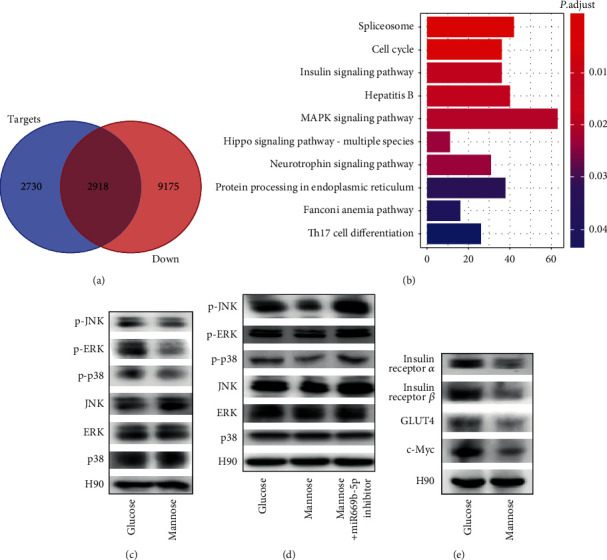


As the significant common pathway plays a key role during D-mannose-mediated adipogenic inhibition, identifying significant common pathways was attempted by GSEA. Crucial common pathways were matched with miR669b-target and D-mannose-downregulated pathways by the Venn method to identify significant common pathways (Figure 4(a)). Key genes serving important roles in significant common pathways were obtained. Based on the criteria mentioned above, the MAPK signaling pathway was identified as the important pathway during the D-mannose regulation (Figure 4(b)). To confirm the result we got, western blot was used to investigate the protein expression levels of the MAPK signaling pathway. The results showed that D-mannose treatment downregulated the expression of p-JNK, p-ERK, and p-p38 proteins compared with the glucose group (Figure 4(c)). And this reduction effect of D-mannose treatment on the MAPK pathway could be reversed by miR669b-5p inhibitor transfection (Figure 4(d)). Moreover, some protein expression levels associated with glucose metabolism were also measured, including insulin receptor *α*, insulin receptor *β*, GLUT4, and c-Myc. The results showed that D-mannose also inhibited the expression levels of these proteins (Figure 4(e)).

## 4. Discussion

Obesity is characterized by fat accumulation [3, 17] and can greatly reduce life expectancy and lead to an increased risk of metabolic diseases [18]. Previously, studies reported that D-mannose improved the metabolism of mice and prevented weight gain, which can be partly explained by D-mannose reducing caloric absorption by the host and increasing the Bacteroidetes-to-Firmicutes ratio in the gut microbiota, a signature associated with the lean phenotype [14]. However, the effect of D-mannose on the differentiation function of adipocyte remains unclear. Therefore, additional studies are necessary to clarify our knowledge of the D-mannose regulation effect on adipocytes since it might help to understand the molecular basis of adipogenesis and identify the new biomarkers or therapeutic targets for the development of antiobesity drugs. Our study is a complement to the previous experiment, which provides an additional scientific explanation of the therapeutic effects of D-mannose on diabetes or obesity.

Alterations in adipose tissue can have profound effects on the development of obesity. Adipose tissue comprises a variety of cells, including adipose-derived stem cells, mature adipocytes, and immune cells [19]. A series of key events occur including adipose-derived stem cell regeneration, proliferation, and adipogenic differentiation, which drive local or systemic obesity and metabolism [20]. Adipogenic differentiation is an ordered multistep process requiring the sequential activation of several groups of transcription factors, including CCAAT/enhancer binding protein (*C/EBP*) gene family, peroxidase proliferator-activated receptor-g (*Pparg*), Krüppel-like factors (*KLFs*), and sterol regulatory element binding protein (*SREBP*) [21, 22]. Hormones and growth factors affect adipogenic differentiation, such as insulin and its receptor [23]. Besides that, little is known about the precise mechanisms of adipogenesis.

MicroRNAs (miRNAs) are a novel group of small (approximately 22 nucleotides) noncoding RNAs that have emerged as important regulators of mRNA expression [18]. Recent studies indicated that microRNAs are involved in the regulatory network of many biological processes, including metabolism and cell differentiation [24, 25]. Several miRNAs were reported to be expressed in adipocytes of mammals and seem to play a role in the regulation of adipogenesis even with potential impact on adipogenesis dysfunctions [18]. So far, some miRNAs including miR-143 [26], miR-210 [27], and miR-204 [28] have been reported to enhance adipogenesis, while other miRNAs such as miR-155 [29], miR-145 [30], and miR-224 [31] showed the inhibition effects. With experimental evidence beginning to elucidate the functional roles of miRNA in adipogenesis and metabolism, the need has become apparent to test miRNA as a viable therapeutic target. However, further investigation is still needed on the role of miRNAs in adipogenesis and the underlying mechanism.

In this study, culture medium with 25 mM D-mannose for adipogenic induction was used to detect the effects of D-mannose on the adipogenic differentiation function of ADSCs. The results showed that D-mannose intervention significantly inhibited the adipogenic differentiation of ADSCs, compared with the glucose intervention. To further explore the mechanisms underlying D-mannose inhibition of ADSC adipogenic differentiation, microarray analysis was performed to detect differences in microRNA expression levels between glucose-treated ADSCs (G-ADSCs) and D-mannose-treated ADSCs (M-ADSCs). And the result showed that miR669b was significantly upregulated by D-mannose compared to the other miRNAs, which was further confirmed by real-time PCR. By transfecting the cells with the microRNA inhibitor mmu-miR-669b-5p, we further confirmed that miR669b was a potential inhibitor of adipogenesis. D-Mannose inhibited the adipogenic differentiation function of ADSCs by increasing the expression of miR669b. Since no previous research has focused on the adipogenic regulation of D-mannose or miR669b, our results may provide a new regulatory target and therapeutic method for the dysregulation of adipogenic differentiation.

The downstream signaling pathway was investigated by GSEA analysis. Crucial common pathways were matched with miR669b-target and D-mannose-downregulated pathways to identify significant common pathways, and the mitogen-activated protein kinase (MAPK) signaling pathway was identified as the important pathway during the D-mannose regulation. The MAPK signaling pathway includes c-Jun N-terminal kinase (JNK), extracellular-signal-regulated kinase (ERK), and p38 MAPK, which are widely expressed and which regulate cell proliferation, differentiation, and death [20]. The research suggested that MAPK signaling including JNK changed immediately before early adipogenesis and activated early adipogenic factors to stimulate adipogenesis [32]. Since lipogenesis plays an important role in insulin resistance by regulating insulin sensitivity and altering the flux of insulin receptor substrates [7, 33], in addition to the exploration of the pathways related to the adipogenic differentiation, we also further investigated the protein expression levels related to the glucose metabolism pathways. p38 MAPK was reported as a signal that may be involved in the activation of GLUT4, a glucose transporter isoform, thus enhancing the insulin response of glucose uptake [34]. The protein product of the protooncogene c-Myc is also a critical downstream effector induced by the MAPK signaling pathways [35]. c-Myc regulates genes involved in the biogenesis of ribosomes and mitochondria and the regulation of glucose and glutamine metabolism, which links altered cellular metabolism to tumorigenesis [36]. In our study, the results of western blot showed that D-mannose intervention inhibited the activation of the MAPK signaling pathway by inhibiting the expression levels of p-JNK, p-ERK, and p-p38 proteins compared with the glucose group. It was reported that miR669b could decrease the expression level of IGF1/IGF1R [37], which was proved to be upstream of the MAPK pathway [38]. However, there is no direct evidence to confirm that miR669b is upstream of the MAPK pathway. We checked the changes of the MAPK pathway after miR669b-5p inhibitor transfection, and the results of western blot showed that the reduction effect of D-mannose treatment on the MAPK pathway could be reversed by miR669b-5p inhibitor transfection, which for the first time proves that miR669b is upstream of the MAPK pathway. Moreover, D-mannose further inhibited the expression of proteins related to glucose metabolisms such as insulin receptor *α*, insulin receptor *β*, GLUT4, and c-Myc, suggesting that D-mannose inhibits the adipogenic differentiation of ADSCs *via* the MAPK pathway and may be further involved in the regulation of glucose metabolism and the inhibition of tumorigenesis.

There were some limitations in this study. We observed the regulation effect of D-mannose on adipogenic differentiation of ADSCs only *in vitro*. However, its regulation of body weight and blood glucose levels *in vivo* still need to be investigated. What is more, it is still a preliminary hypothesis that D-mannose inhibited the MAPK signaling pathway and thus suppressed the activation of GLUT4 and the insulin response of glucose uptake. Further studies still need to be carried out to explore the potential mechanism.

## 5. Conclusions

In conclusion, our results indicated that D-mannose inhibits adipogenic differentiation of ADSCs *via* the miR669b/MAPK signaling pathway. To the best of our knowledge, this is the first study to report the effect of D-mannose on adipogenic differentiation of ADSCs. Moreover, we also found miR669b as a new regulatory target during the regulation of D-mannose on adipogenic differentiation. Our results might help to understand the molecular mechanisms of adipogenesis and identify the new therapeutic targets for the development of antiobesity drugs.

## Figures and Tables

**Table 1:** The primers used in this experiment.

	Sense	Antisense
*Bglap*	5-CGCTACCTGTATCAATGGCTGG-3	5-CTCCTGAAAGCCGATGTGGTCA-3
*Alpl*	5-ATGGGATGGGTGTCTCCACA-3	5-CCACGAAGGGGAACTTGTC-3
*Pparg*	5-CTCCTATTGACCCAGAAAGC-3	5-GTAGAGCTGAGTCTTCTCAG-3
*Fabp4*	5-GTCCAGGCTGGAATGCAGTG-3	5-CACACAGACGTACAGAGTGG-3
*GAPDH*	5-AGCCGCATCTTCTTTTGCGTC-3	5-TCATATTTGGCAGGTTTTTCT-3

## Data Availability

All data are provided in full in the results of our manuscript, and the necessary detail can be provided by the corresponding authors on request.
